# ADP-Ribose and oxidative stress activate TRPM8 channel in prostate cancer and kidney cells

**DOI:** 10.1038/s41598-018-37552-0

**Published:** 2019-03-11

**Authors:** Ercan Baş, Mustafa Nazıroğlu, László Pecze

**Affiliations:** 1 Department of Urology, Faculty of Medicine, Suleyman Demirel University, Isparta, Turkey; 2 Neuroscience Research Center, Suleyman Demirel University, Isparta, Turkey; 3 Drug Discovery and Development Research Group, BSN Health, Analysis and Innovation, Goller Bolgesi Teknokenti, Isparta, Turkey; 4 Independent Scientist, Basel, Switzerland

## Abstract

Activation of TRPM8 channel through oxidative stress may induce Ca^2+^ and pro-apoptotic signals in prostate cancer and kidney cells. The aim of this study was to evaluate activation of TRPM8 can increase apoptosis and oxidative stress in the prostate cancer (Du145^M8^), TRPM8 knock out (Du 145^M8KO^), transfected (HEK293^TM8^) and non-transfected human kidney (HEK293) cells. Intracellular Ca^2+^ responses to TRPM8 activation were increased in the Du145^M8^ and HEK293^TM8^ cells from coming cumene hydrogen peroxide (CHPx), menthol, ADP-Ribose (ADPR), but not in the HEK293 and Du 145^M8KO^ cells. The intracellular Ca^2+^ responses to both ADPR and CHPx were totally inhibited by the thiol cycle antioxidant glutathione, and TRPM8 blockers (N-(p-amylcinnamoyl)anthranilic acid and capsazepine). Apoptosis, Annexin V, mitochondrial membrane depolarization, intracellular ROS, caspase 3 and 9 values were increased through TRPM8 activation in the Du 145^M8^ but not in the Du 145^M8KO^ and non-transfected HEK293 cells by CHPx and hydrogen peroxide. In conclusion, apoptotic and oxidant effects on the cells were increased activation of TRPM8 by oxidative stress and ADPR. Activation of TRPM8 through oxidative stress and ADPR in the cells could be used as an effective strategy in the treatment of prostate cancer cells.

## Introduction

Oxidative stress occurs during the physiological functions such as phagocyte activity and mitochondrial function. The oxidative stress is controlled by the antioxidants such as glutathione (GSH) and glutathione peroxidase (GSH-Px). GSH as a member of thiol cycle antioxidants endogenously synthesized all mammalian cells and it has several physiological functions such as antioxidant defense, inhibition of prostate cancer and transport of cysteine^1,2^. GSH and N acetyl cysteine (NAC) treatments as a member of thiol redox system, induced transient receptor (TRP) melastatin 2 (TRPM2) and 8 (TRPM8) channel inhibitor roles^3–6^. ADP-Ribose (ADPR) is synthesized in the nucleus beta nicotinamide adenine dinucleotide by activation CD38 enzyme through hydrogen peroxide (H_2_O_2_) production^7,8^. The H_2_O_2_ has been using for investigation of oxidative stress dependent TRP channel activations such as TRPM2 and TRPV1^7–9^. The TRPM8 channel is activated by cold and menthol^10,11^. However, there is no report ADPR and H_2_O_2_ dependent activation of TRPM8 in the prostate cancer and human embryonic kidney cells 293 (HEK293) cells.

Intracellular free calcium ion ([Ca^2+^]_i_) concentration is a major intracellular second messenger factor that regulates many physiological and pathophysiological functions including cell migration^12,13^. Apoptosis, proliferation, differentiation and migration in cells are controlled by the Ca^2+^ signaling pathways. Prostate cancers are a most common diagnosis in men. It is also well known that an increase of [Ca^2+^]_i_ concentration involved in prostate cancer carcinogenesis and in metastasis development^14^. The Ca^2+^ passes the cell membranes through different cation channels including TRP channels. As a member of the TRP superfamily, TRPM8 channel, changes in its expression level is involved in the etiology of prostate cancers and it seems to be one of the most promising potential drug target channels in the treatment of prostate cancers^15^. Androgen-dependent expression of TRPM8 increases in both benign prostate hyperplasia and in prostate carcinoma cells^15,16^. Involvement of transmembrane domains-isoforms of TRPM8 in the mitochondria of keratinocyte cells for the regulating [Ca^2+^]_i_ concentration was recently reported^17^. In addition, an increase of [Ca^2+^]_i_ concentration through menthol activation of TRPM8 channels in the prostate cancer cells induced increase the rate of mitochondrial oxidative stress, resulting apoptosis of the cancer cells^18^. Hence, activation of TRPM8 through oxidative stress may induce pro-apoptotic signals in prostate cancer cells, but it remains unclear.

To our knowledge, there is no report on the oxidative stress and ADPR dependent activation of TRPM8 channels in TRPM8 positive androgen insensitive prostate cancer (Du 145^M8^) and overexpressing human TRPM2 channel HEK293 (HEK293^TM8^) cells. Therefore, we propose that investigation of the involvement of oxidative stress in the TRPM8 activation might represent two of the mechanisms controlling up-regulation of mitochondrial oxidative stress, apoptosis and [Ca^2+^]_i_ concentration in the Du 145^M8^ and HEK293^TM8^ cells.

## Results

### Oxidative stress activates TRPM8 in the Du 145^M8^ cells

**Figure 1:**
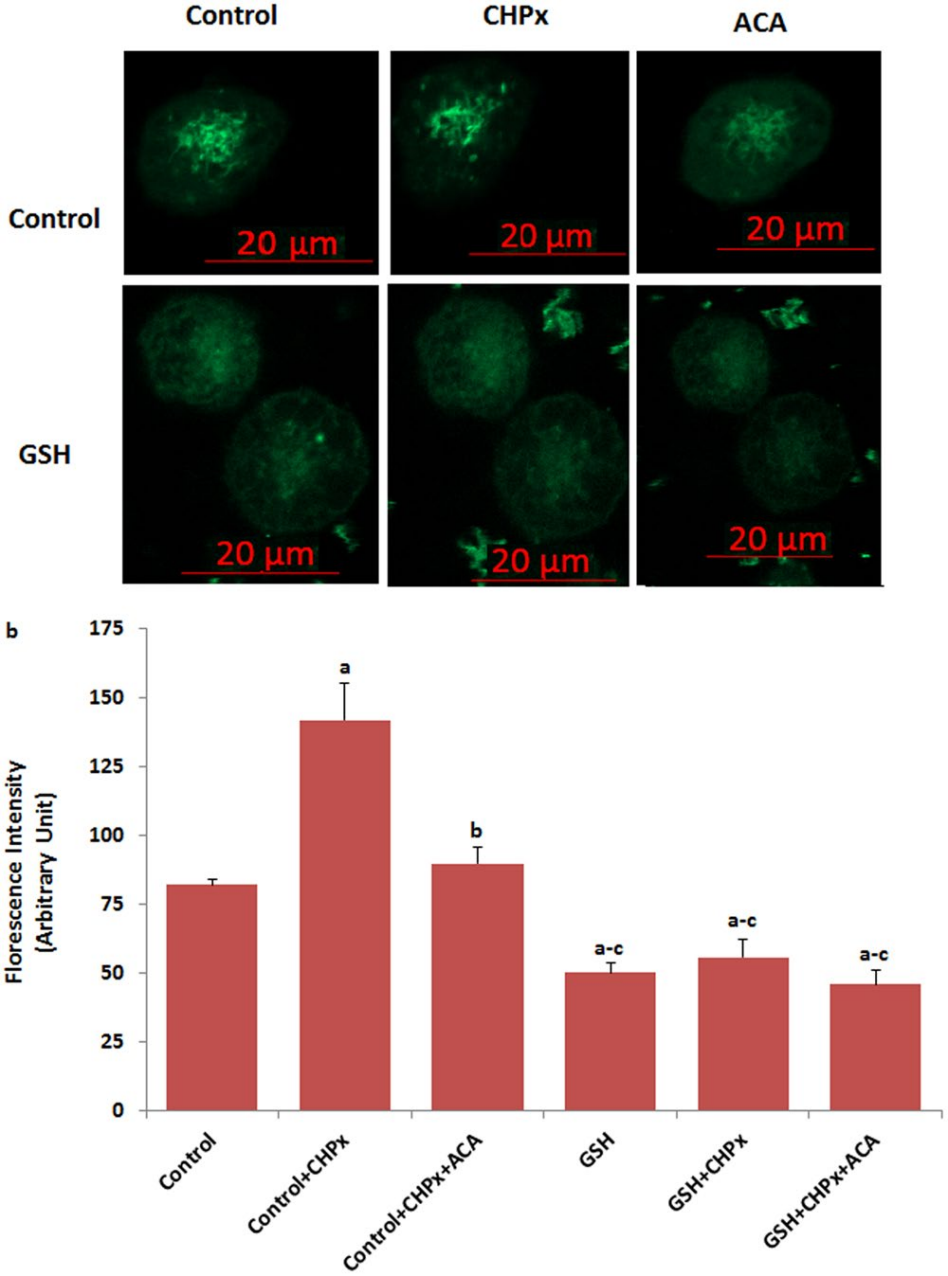
Activation of TRPM8 in the Du 145^M8^ cells by oxidative stress. (mean ± SD). The cells were stained with Fluo-3 calcium dye and mean ± SD of fluorescence in 15 mm^2^ of cell as arbitrary unit are presented; n = 10–20 independent experiments. In GSH experiments, the cells were pretreated with GSH (10 mM for 2 hours). The cells were extracellularly stimulated by cumene hyroperoxide (CHPx and 1 mM for 5 min) but they were extracellularly inhibited by ACA (25 μM for 10 min). The samples were analyzed by the laser confocal microscopy fitted with a 40× oil objective. The scale bar was 20 µm. Representative images and fluorescence intensities of the CHPx, ACA and GSH effect on the TRPM8 activation in the laser confocal microscope analyses are shown in (**a**,**b**) respectively. (^a^p ≤ 0.001 versus control. ^b^p ≤ 0.001 versus control + CHPx group. ^c^p ≤ 0.001 versus control + CHPx + ACA group).

As the first step in the current study whether activation of TRPM8 channel is related to oxidative stress (cumene hyroperoxide, CHPx) activator and menthol, the influences of the channel on Ca^2+^ fluorescence intensity in the Du 145 cells were investigated by using the activators and inhibitors (thiol cycle antioxidant GSH and TRPM8 channel blocker [N-(p-amylcinnamoyl)anthranilic acid (ACA)]. The confocal microscope images (Fig. 1a) and columns (Fig. 1b) of Ca^2+^ fluorescence intensity in Du 145^M8^ are presented in Fig. 1. The Ca^2+^ fluorescence intensity was increased in the cells by CHPx stimulations. On the other word, the Ca^2+^ fluorescence intensity was significantly (p ≤ 0.001) higher in the control + CHPx groups as compared to control. However, the Ca^2+^ fluorescence intensity was markedly (p ≤ 0.001) decreased in the control + CHPx + ACA group as compared to the CHPx group by the ACA treatment. This increase in Ca^2+^ fluorescence intensity was totally prevented by pretreatment with GSH and the Ca^2+^ fluorescence intensity was markedly (p ≤ 0.001) lower in the GSH, GSH + CHPx and GSH + CHPx + ACA than in the control + CHPx and control + CHPx + ACA groups.

### Oxidative stress has no TRPM8 activation in the absence of TRPM8 and extracellular Ca^2+^ in the Du 145^M8^ and Du 145^M8KO^ cells

**Figure 2:**
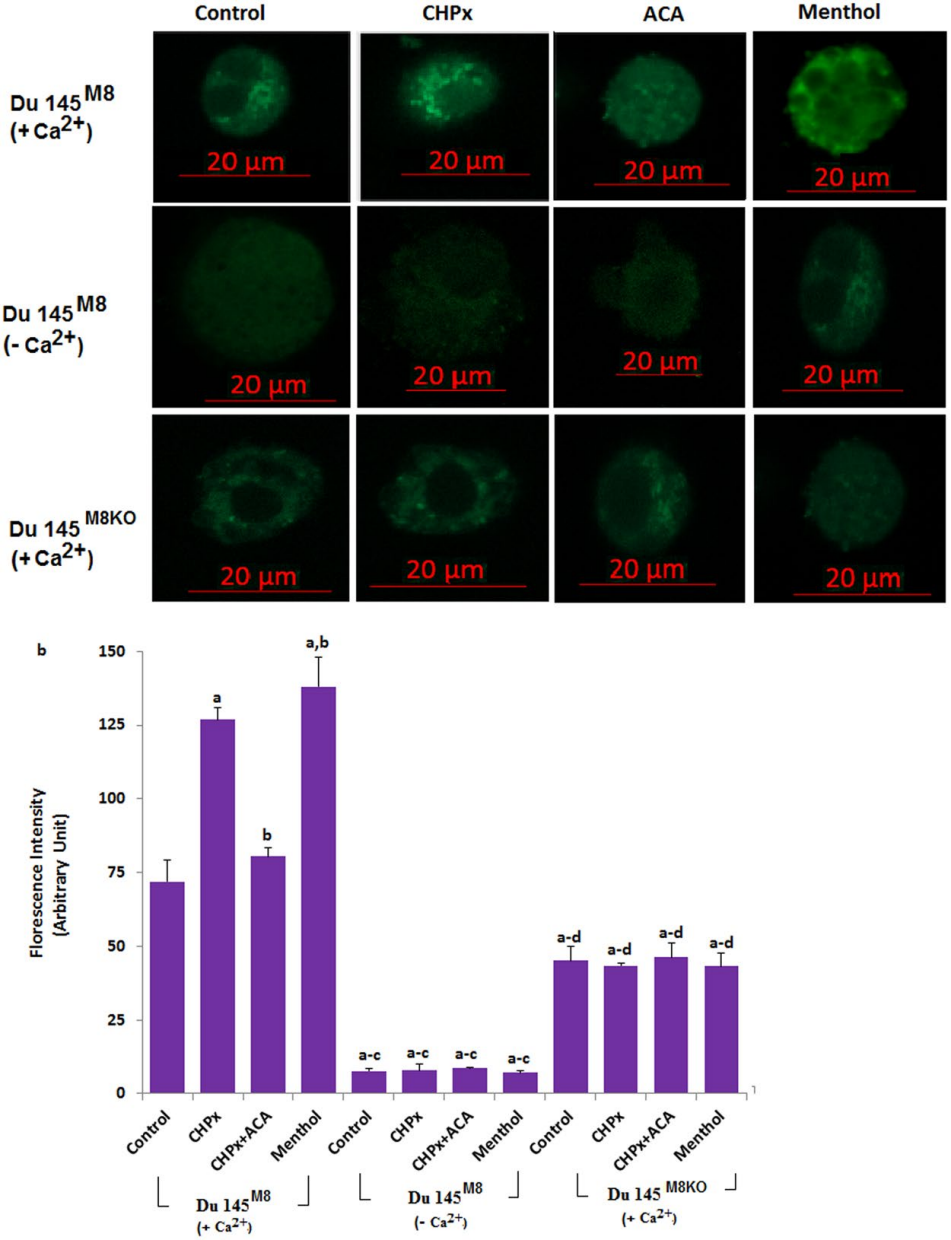
There is no activation of TRPM8 in the Du 145^M8^ and Du 145^M8KO^ cells without extracellular Ca^2+^ by oxidative stress (CHPx) and menthol. (mean ± SD). (+Ca^2+^, Ca^2+^-containing extracellular buffer; −Ca^2+^, Ca^2+^-free buffer). The cells were stained with Fluo-3 calcium dye and mean ± SD of fluorescence in 15 mm^2^ of the cells as arbitrary unit are presented; n = 10–20 independent experiments. The Du 145^M8^ and Du 145^M8KO^ cells with +Ca^2+^and −Ca^2+^ buffers in the TRPM8 experiments were stimulated by CHPx (1 mM for 10 min) but they were inhibited by ACA (25 μM for 10 min). The samples were analyzed by the laser confocal microscopy fitted with a 40× oil objective. The scale bar was 20 µm. Representative images and fluorescence intensities of the CHPx, ACA and menthol effects on the TRPM8 activation in the laser confocal microscope analyses are shown in Fig. 1a,b, respectively. (^a^p ≤ 0.001 versus control. ^b^p ≤ 0.001 versus control + CHPx group. ^c^p ≤ 0.001 versus control + CHPx + ACA group. ^c^p ≤ 0.001 versus Du 145^M8^ (without Ca^2+^ groups).

After observation of oxidative stress dependent activation of TRPM8 in the cells, we tested the effects of absence or presence of extracellular Ca^2+^ (+Ca^2+^, Ca^2+^-containing extracellular buffer; −Ca^2+^, Ca^2+^-free buffer) or deletion of TRPM8 (Du 145^M8KO^) in the Ca^2+^ fluorescence intensity of Du 145^M8^ cells. Du 145^M8KO^ cells, which do not express TRPM8 channels^18^, showed no detectable TRPM8 response-induced Ca^2+^ fluorescence intensity (Fig. 2a,b). Addition of CHPx and menthol in the presence of Ca^2+^ led to a significant increase in the Ca^2+^ fluorescence intensity in the Du 145^M8KO^ cells, which was decreased by the addition of ACA, the TRPM2 channel specific inhibitor (Fig. 2a,b). In contrast, CHPx and menthol treatments induced no increase in the Ca^2+^ fluorescence intensity level in the absence of Ca^2+^ (Fig. 2a,b). Furthermore, the Ca^2+^ fluorescence intensity increases were not observed in the absence of TRPM8 in the Du 145^M8KO^ cells. These results exclude the Ca^2+^ release from intracellular organelles such as the endoplasmic reticulum and mitochondria and more importantly, for the first time, demonstrate the existence of a specific mechanism for Ca^2+^ influx involving TRPM2 channels.

### TRPM2 blocker (ACA) inhibits the ADPR -induced TRPM8 currents in the Du 145^M8KO^ cells

**Figure 3:**
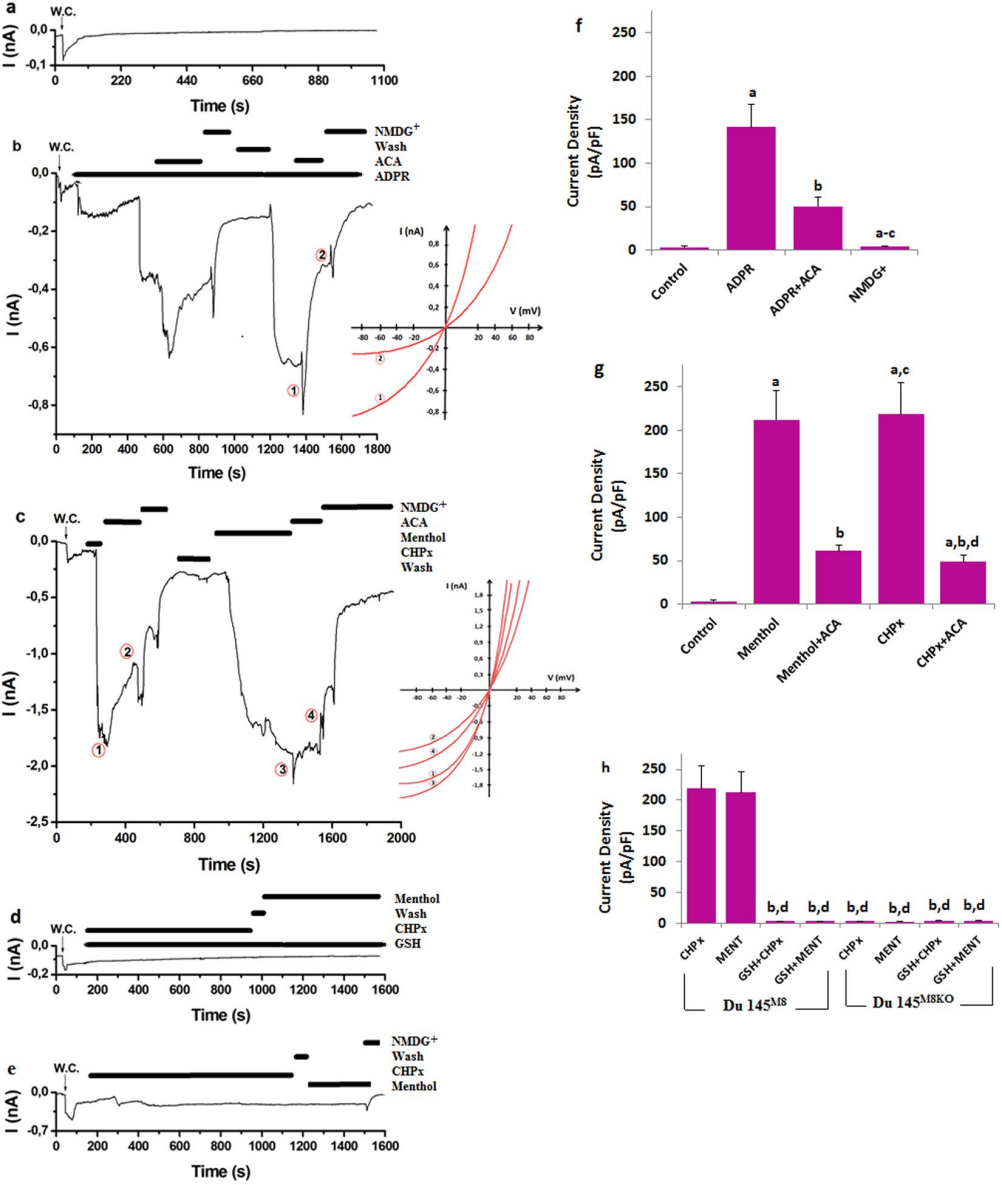
Effect of oxidative stress (CHPx) and ADPR on the TRPM8 current densities (pA/pF) in the Du 145^M8^ and Du 145^M8KO^ cells. (mean ± SD and n = 3). The TRPM8 currents in the Du 145^M8^ and Du 145^M8KO^ cells were induced either by intracellular ADPR (1 mM in patch-pipette) or extracellular CHPx (10 mM) and 0.1 mM menthol, but they were blocked by extracellular ACA (25 μM) in the patch-chamber. Intracellular GSH (2 mM) was given to the cells in the patch pipette. W.C.: Whole cell. (**a**) Control: Original recordings from control neuron. (**b**) ADPR group. (**c**) Menthol and CHPx group. (**d**) GSH group. (**e**) the Du 145^M8KO^ cells group. The (**f**–**h**) were currents densities of (**b**–**d**) +e patch clamp records, respectively. (^a^p ≤ 0.001 versus control. ^b^p ≤ 0.001 versus ADPR and menthol groups. ^c^p ≤ 0.001 versus ADPR + ACA and menthol + ACA groups. ^d^p ≤ 0.001 versus CHPx group).

ADPR is synthesized in the nucleus beta nicotinamide adenine dinucleotide by activation CD38 enzyme through extracellular H_2_O_2_ production^19,20^. As a member of TRP superfamily, TRPM2 channel is activated by ADPR^19,20^ but there is no report on the ADPR-induced TRPM8 in cells. Therefore, we firstly tested involvement of ADPR on the TRPM8 activation in the Du 145 cells. TRPM8 channel in the patch-clamp experiments was gated in the Du 145^M8^ cells by ADPR (1 mM in the patch-pipette), although they were reversibly blocked by ACA and NMDG^+^ (replacement of Na^+^) (Fig. 3b). There were no currents in the absence of the TRPM8 agonists (ADPR, CHPx and menthol) and antagonists (and ACA) (Fig. 3a). Treatment of wild type (Du 145^M8^) cells with the 25 μM ACA as a TRPM2 channel inhibitor, strongly suppressed ADPR-induced current densities (Fig. 3b,c). On the other word, the current densities in the cells were significantly higher in the control + ADPR group compared with the control group (p ≤ 0.001); however, the current density of TRPM8 was significantly (p ≤ 0.001) lower in the control + ADPR + ACA group than in the control + ADPR group (Fig. 3b,f).

The H_2_O_2_ has been using for investigation of oxidative stress dependent TRP channel activation such as TRPM2 and TRPV1^7,9^. To further investigate the relative contribution of oxidative stress in the TRPM8 activation, the effect of CHPx was studied in the TRPM8 present (Du 145^M8^) and knockout (Du 145^M8KO^) prostate cancer cells (Fig. 3c–e). In addition, we used specific agonist of TRPM8 (menthol) as positive control records. The current densities in the neurons were increased in CHPx and menthol groups (Fig. 3g), and they were decreased in the CHPx + ACA and menthol + ACA groups by the ACA treatments (p ≤ 0.001) (Fig. 3g). Hence, these effects of CHPx and menthol were partially abolished by ACA.

In patch clamp experiment, we also tested the role of antioxidant GSH and deletion of TRPM8 on the TRPM8 activation in the Du 145^M8^ cells. The menthol and CHPx-induced currents were completely blocked in the presence of intracellular GSH (2 mM in the patch pipette) (Fig. 3d) and deletion of TRPM8 (Fig. 3e). The current densities were markedly (p ≤ 0.001) lower in the Du 145^M8^ + GSH, Du 145 GSH + menthol, Du 145^M8^ + GSH + CHPx and Du 145^M8KO^ groups than in the Du 145^M8KO^ + menthol and Du 145^M8KO^ + CHPx groups.

These results clearly indicated that oxidative stress induced excessive Ca^2+^ influx through the TRPM8 channel. However, the oxidative stress-induced TRPM8 currents through ROS production modulation were decreased by treatment with the antioxidant (GSH).

### Hydrogen peroxide induces TRPM8-dependent increase of Ca^2+^ fluorescence intensity in the HEK293 cells overexpressing human TRPM8 channel (HEK293^TM8^) cells

**Figure 4:**
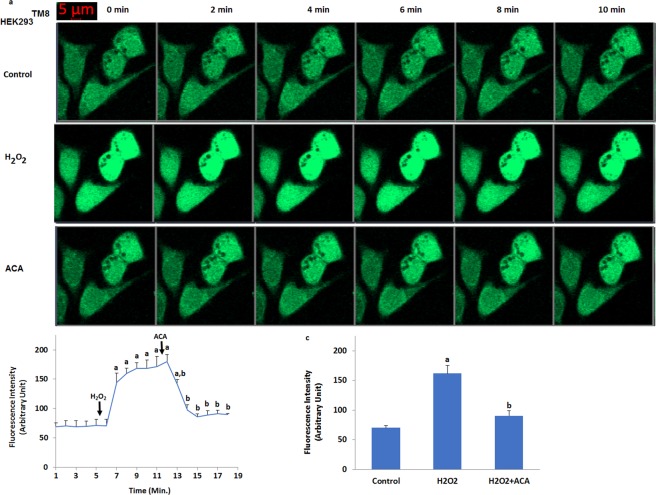
Activation of TRPM8 in the non-transfected (HEK293) and transfected (HEK293^TM8^) human HEK293 cells by hydrogen peroxide (H_2_O_2_). (mean ± SD). The cells were stained with Fluo-3 calcium dye and mean ± SD of fluorescence in 15 mm^2^ of the cells as arbitrary unit are presented; n = 10–20 independent experiments. The HEK293^TM8^ cells were stimulated by H_2_O_2_ (1 mM for 10 min) but they were inhibited by ACA (25 μM for 10 min). The samples were analyzed by the laser confocal microscopy fitted with a 40× oil objective. The scale bar was 5 µm. Representative images (**a**), line (**b**) and column (**c**) of fluorescence intensities of the H_2_O_2_ and ACA on the TRPM8 activation in the laser confocal microscope analyses are shown in Figs a–c, respectively. (^a^p ≤ 0.001 versus control. ^b^p ≤ 0.001 versus H_2_O_2_ group).

**Figure 5:**
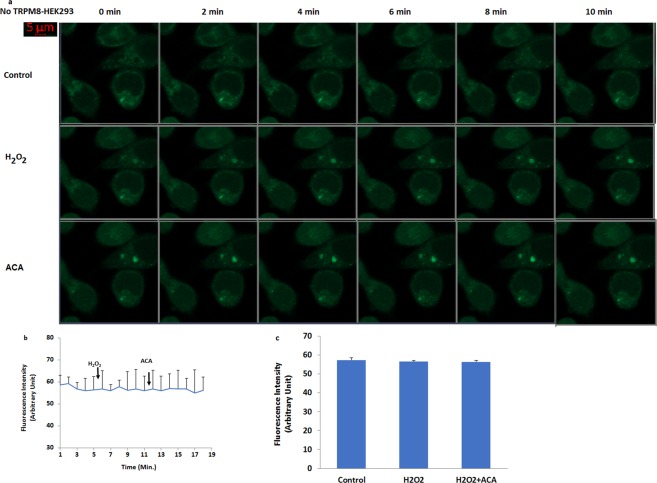
No activation of TRPM8 in the HEK293 without overexpressing human TRPM2 channel by hydrogen peroxide (H_2_O_2_). (mean ± SD). The cells were stained with Fluo-3 calcium dye and mean ± SD of fluorescence in 15 mm^2^ of the cells as arbitrary unit are presented; n = 10–20 independent experiments. The HEK293 cells were stimulated by H_2_O_2_ (1 mM for 10 min) but they were inhibited by ACA (25 μM for 10 min). The samples were analyzed by the laser confocal microscopy fitted with a 40× oil objective. The scale bar was 5 µm. Representative images, line and column of fluorescence intensities of the H_2_O_2_ and ACA on the TRPM8 activation in the laser confocal microscope analyses are shown in Figs a–c, respectively.

After observation of oxidative stress dependent activation of TRPM8 in the cells, we tested effects of oxidative stress (H_2_O_2_) on the fluorescence intensity (Figs 4 and 5) through TRPM8 activation in the HEK293^TM8^ cells. It is well known that TRPM2 channel is activated within 2–5 minutes in different cell lines by oxidative stress and ADPR^19,20^. Similarly, we observed activation of TRPM8 in HEK293^TM8^ cell within 2–5 minutes by ADPR and H_2_O_2_ (Fig. 4b). There was no increase in the fluorescence intensity of Ca^2+^ in the control HEK293^TM8^ cells within 6 minutes (Fig. 4b). The intensity was markedly (p ≤ 0.001) increased in the cell by H_2_O_2_ (Fig. 4b,c), although it was decreased TRPM2 channel blocker (ACA). However, HEK293 cells, which do not express TRPM8 channels, showed no detectable TRPM8 response-induced Ca^2+^ fluorescence intensity through activation of TRPM8 by the H_2_O_2_ stimulation (Fig. 5a,b) and the fluorescence intensity levels did not change in the control, H_2_O_2_ and ACA groups, statistically. It is well known that several TRP channels such as TRPM2 and TRPM7 can be activated by oxidative stress^12^. These results in the TRPM8 expressing the HEK293^TM8^ cells exclude involvement of oxidative stress dependent activated other TRP channels and more importantly, for the first time, demonstrate the existence of a specific mechanism for oxidative stress-induced Ca^2+^ influx involving TRPM8 channels.

### ADPR and hydrogen peroxide induce TRPM8-dependent increase of [Ca^2+^]_i_ concentration in the HEK293 cells overexpressing human TRPM8 channel (HEK293^TM8^) cells: Single cell patch clamp records

**Figure 6:**
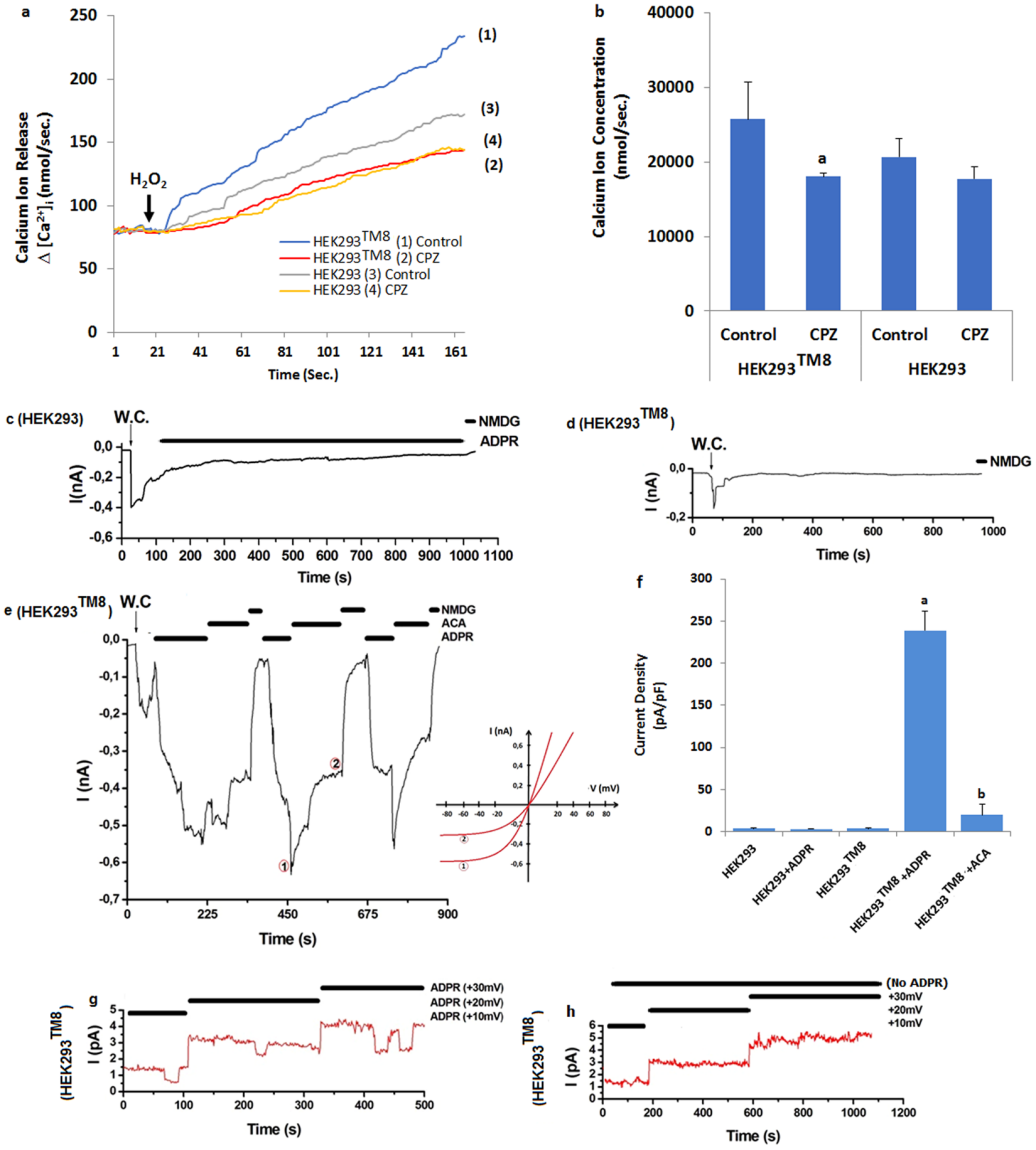
Effects of ADPR and oxidative stress (H_2_O_2_) on the TRPM8 current densities (pA/pF) in the HEK293 and HEK293^TM8^ cells. (mean ± SD and n = 6). Fura-2-loaded the HEK293 and HEK293^TM8^ cells were stimulated with H_2_O_2_ (1 mM) in the presence of normal extracellular calcium (1.2 mM) for 160 seconds. The results were expressed as lines (**a**) and columns (**b**). In patch-clamp experiments, the TRPM8 currents in the HEK293 and HEK293^TM8^ cells were induced by intracellular ADPR (1 mM in patch-pipette), but they were blocked by extracellular ACA (25 μM) in the patch-chamber. W.C.: Whole cell. (**c**) Original recordings from HEK293 with ADPR stimulation. (**d**) Original recordings from HEK293^TM8^ without ADPR stimulation. (**e**) Original recordings from HEK293^TM8^ with ADPR stimulation and ACA inhibition. The f was current densities of e patch clamp records. (**g**) ADPR. Single cell records from HEK293^TM8^ cells. (**g**) No ADPR. Control single cell records from HEK293^TM8^ cells. (^a^p ≤ 0.001 versus HEK293^TM8^ group. ^b^p ≤ 0.001 versus HEK293^TM8^ + ADPR group).

After observation of the oxidative stress dependent increase of TRPM8 in the cells, we tested the effects of ADPR and oxidative stress (H_2_O_2_) on the Ca^2+^ fluorescence intensity in the overexpressing human TRPM8 channel (HEK293^TM8^) cells, we wanted further confirms the results of measurements of [Ca^2+^]_i_ concentration via Fura-2 analyses and current density via patch-clamp analyses. Again, the HEK293 cells, which do not express TRPM8 channels, showed no detectable TRPM8 response-induced [Ca^2+^]_i_ concentration (Fig. 6a,b) current density (Fig. 6c,f) through activation of TRPM8 by the H_2_O_2_ and ADPR stimulations. Induction of TRPM2 expression using a transfection system, however resulted in decrease ADPR and oxidative stress-sensitive [Ca^2+^]_i_ concentration (Fig. a,b) and current density (Fig. 6e,f) through ACA treatment. In addition, we observed ADPR dependent activation in the single channel (inside out) patch clamp records (Fig. 6g). However there was no the single channel currents in the absence of ADPR (Fig. 6h). The single channel results exclude the involvement of second messengers for the activation of TRPM8 via oxidative stress and ADPR. On the other word, it is more importantly, for the first time, demonstrate the existence of a specific mechanism as a TRPM2 channel for Ca^2+^ involving TRPM8 channels.

### Involvement of TRPM8 in oxidative stress-induced Du 145^M8^ cell apoptosis and ROS generation

**Figure 7:**
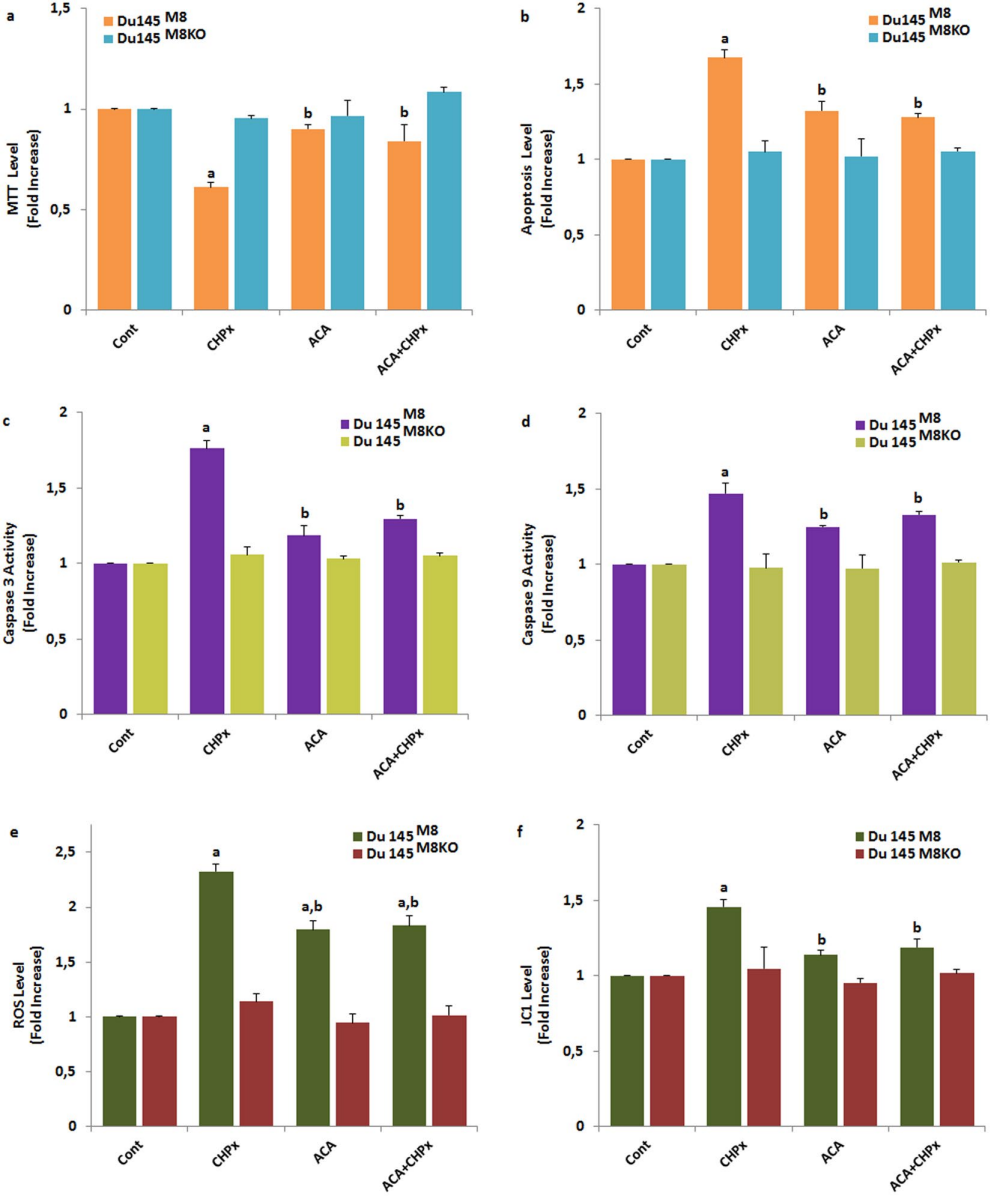
Effect of CHPx (1 mM) and ACA on the cell viability (MTT) (**a**), apoptosis (**b**), caspase 3 (**c**), caspase 9 (**d**), intracellular ROS production (**e**) and mitochondrial membrane depolarization (JC1) (**f**) levels in the Du 145 wild type (Du145^M8^) and Du 145-knockout (Du145^M8KO^) cells. (mean ± SD and n = 3). The TRPM8 currents in the Du 145^M8^ cells were induced by with CHPx (1 mM for 10 min) but they were blocked by extracellular ACA (25 μM for 10 min). Then, cells in the four groups were further stimulated by CHPx (1 mM). (^a^p ≤ 0.001 versus control group. ^b^p ≤ 0.001 versus CHPx group).

The excessive Ca^2+^ entry is an important source of ROS that induce cell death and ROS is known to activate several TRP channels. Next, we examined whether TRPM8 were attenuated in ROS-induced apoptosis, cell viability and caspase activation by determining the effects of ACA as a TRPM8 inhibitor, on oxidative stress-induced prostate cancer cell apoptosis and generation of ROS. The results of MTT (Fig. 7a), apoptosis (Fig. 7b), caspase 3 (Fig. 7c), caspase 9 (Fig. 7d), intracellular ROS production (Fig. 7e) and mitochondrial membrane depolarization (JC1) (Fig. 7f) in the four groups of Du 145^M8^ and Du 145^M8KO^ cells are shown in Fig. 7. Compared with control, CHPx treatment in the Du 145^M8KO^ cells increased the levels of apoptosis, ROS, JC1, caspase 3 and 9 (p ≤ 0.001), although MTT levels in the cells was decreased by the CHPx treatment (Fig. 7a) (p ≤ 0.001). However, there were no differences in the values in the four groups of Du 145^M8^ and Du 145^M8KO^ cells. More importantly, we found ACA reduced the levels of apoptotic cells through the decrease of the ROS, JC1, caspase 3 and 9 values and increase of the MTT levels in the cells (p ≤ 0.001). However, Du 145^M8KO^ cells, which do not express TRPM8 channels, showed no detectable TRPM8 response-induced apoptosis, ROS, JC1, caspase 3 and 9 through activation of TRPM8 by the CHPx stimulation (p ≥ 0.05). Our data suggested that the involvement of TRPM8 channels on the oxidative stress-induced apoptosis in the cancer cells, because oxidative stress**-**induced apoptosis, which could be inhibited by TRPM8 blocker (ACA) treatment.

### Involvement of TRPM8 in fluorescence intensity of Annexin V (aV), mitochondrial membrane depolarization (JC1) and intracellular ROS production levels in the non-transfected (HEK293) and transfected human HEK293 (HEK293^TM8^) cells

**Figure 8:**
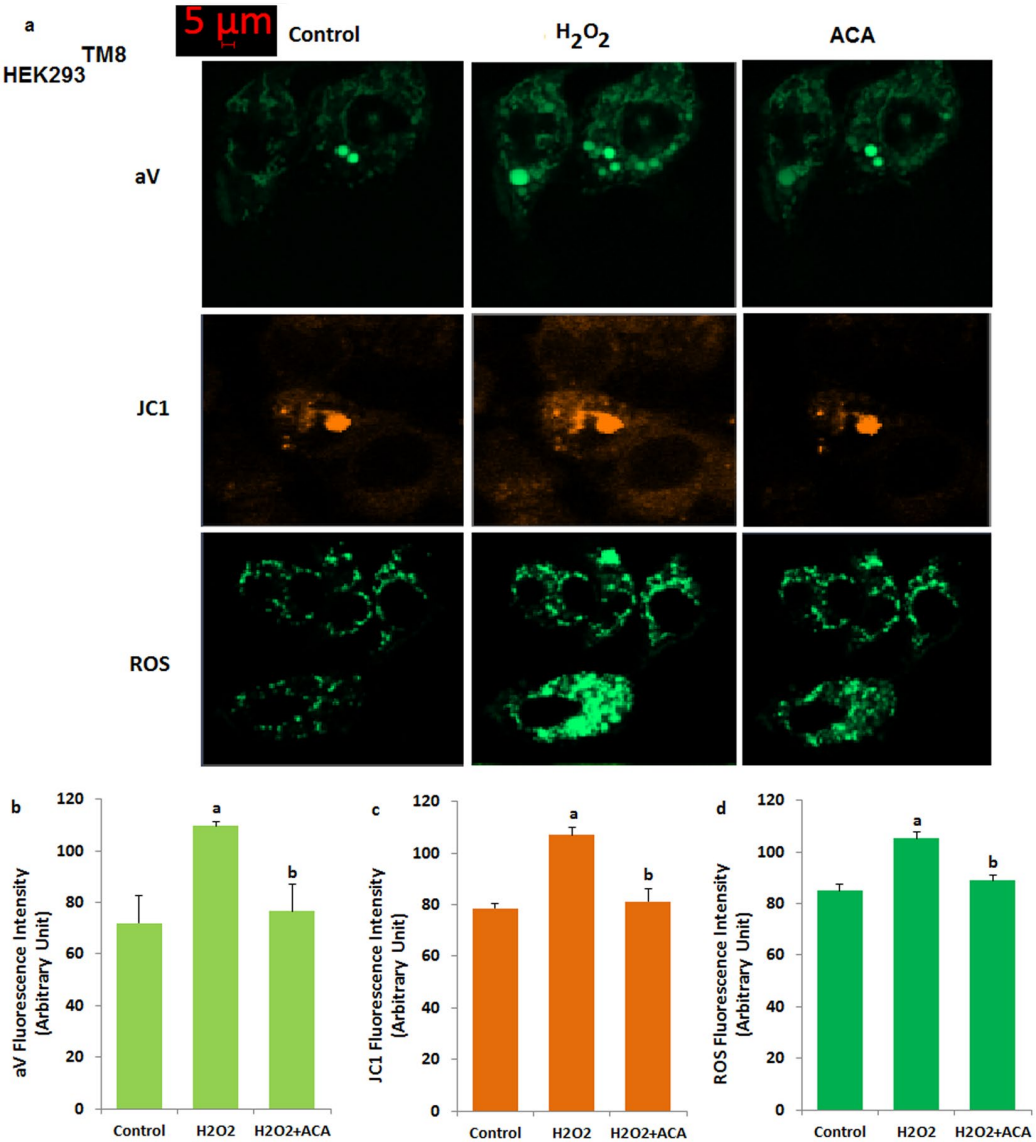
Effect of H_2_O_2_ and ACA on apoptosis (Annexin V, aV) (**a**,**d**), mitochondrial membrane depolarization (JC1) (**a**,**c**) and intracellular ROS production (**a**,**d**) fluorescence intensity levels in the transfected HEK293 (HEK293^TM8^) cells. (mean ± SD and n = 10–20). The cells were stimulated with H_2_O_2_ (1 mM for 10 min), but they were blocked by extracellular ACA (25 μM for 10 min). Then, the cells in the four groups were further stimulated by H_2_O_2_ (1 mM). (^a^p ≤ 0.001 versus control group. ^b^p ≤ 0.001 versus H_2_O_2_ group).

We further studied certain mitochondrial oxidative stress-related apoptosis (aV) induced by the H_2_O_2_. The fluorescence intensity of aV (a and b), JC1 (a and c) and ROS (a and d) results are shown in Fig. 8. The aV, JC1 and ROS levels were increased by the H_2_O_2_ incubation. On the other word, the aV, JC1 and ROS levels were markedly (p ≤ 0.001) higher in the H_2_O_2_ group as compared to control. In addition, the increased aV, ROS and JC1 levels were markedly (p ≤ 0.001) decreased in the ACA and ACA + H_2_O_2_ groups by the ACA treatment. However, there were no differences on the aV, JC1 and ROS values in the control, H_2_O_2_ and H_2_O_2_ + ACA groups of non-transfected HEK293 cells (Data are not shown).

## Discussion

In the current study, we found that oxidative stress and ADPR treatments could induce the TRPM8 activations resulting in the overload Ca^2+^ entry, apoptosis, and mitochondrial oxidative stress. More importantly, we found that GSH could protect the Du 145^M8^ prostate cancer cells from oxidative stress-induced apoptosis via maintaining the intracellular Ca^2+^ homeostasis as well as down-regulating mitochondrial oxidative stress pathway. The major findings of this study are that TRPM8 channel is separately activated in the prostate cancer cells by ADPR and oxidative stress and its sensitivity enhance to ROS.

There is debating evidence obtained from the prostate cancer and human kidney cells, that TRPM8 channel activation is associated with production of oxidative stress^17,18,21^. Indeed, H_2_O_2_ stimulation induced functional changes on the TRPM8 in the urothelium cell of elderly subject and human lung epithelial cells, although the changes were reduced by NAC treatments^20^. However, conflicting report is also presented on the subject and the TRPM8 channel was not activated in urothelium bladder cells by 1 mM H_2_O_2_^21^. In general, induction of oxidative stress as a mechanism that may contribute to the antitumor induction effect has been gaining acceptance^15^. Most of chemotherapeutic agents induce excessive ROS production for killing the cancer cells^14^. It is well known that an increase in [Ca^2+^]_i_ concentrations through activation of TRP channels such as TRPM2 and TRPV1 induces an increase of intracellular mitochondrial ROS production^22,23^. However, GSH as a member of thiol cycle antioxidants has been shown to inhibit CHPx-evoked increased in cell viability and decreases in intracellular levels of ROS and apoptosis^13,14,23^. GSH has been also reported to prevent completely ADPR and CHPx-evoked TRPM2 and TRPV1 channel activations^13,14,23^. Thus, the pro-apoptotic effects of oxidative stress in the cancer cells, including prostate cancer cells seem to be dependent on one single mechanism, e.g., the ability of TRPM8 activation to generate oxidative stress. We have recently identified the primary role of menthol dependent, but not oxidative stress TRPM8 activation in the Du 145 cells^18^. GSH and NAC treatments as two members of thiol redox system, induced TRPM2 and TRPM8 channel inhibitor roles through inhibition of oxidative stress in different cell lines^3–6^ . Of interest for the present discussion is the finding that ADPR and CHPx-evoked TRPM8 currents were completely abated by intracellular GSH treatment. These findings imply that oxidative stress directly gates TRPM8, but rather probably exerts this action indirectly via the generation ADPR in DNA damage of nucleus by oxidative stress byproducts that eventually target the channel in the prostate cancer cells, through the direct formation of intracellular ROS^4^.

In the current study, we observed increased levels of apoptosis, caspase 3, caspase 9, mitochondrial membrane depolarization and ROS values through activation of TRPM8 channel in the Du 145^M8^ cells, but not Du Du 145^M8KO^ cells by CHPx and ADPR, although the values were decreased in the cells by the GSH treatment. During the treatment of tumor cells including prostate cancer cells, increase of mitochondrial oxidative stress through activation of TRPM8 channels and mitochondrial dysfunction has been suggested to account in cancer cells the induction of apoptosis^24,25^. Mitochondrial oxidative stress and apoptosis in human epithelial prostate cancer cells were induced by suppression of TRPM8 isoforms^17^, through alterations in mitochondrial membrane depolarization and ATP production^26^, which leads to oxidative phosphorylation through the electron transport chain and hence the formation of JC1^27^. Thus, induction of apoptosis through overload Ca^2+^ entry by oxidative stress probably lead to the increase of this toxic protein aggregates inhibiting cancer cell survival. It has been reported that Ca^2+^ entered from the cytosol during mitochondrial stress accumulates in the mitochondria and mediates the excessive apoptosis through activation of caspase 3 and 9^18^. ROS generation activates both survival and death signaling, depending upon the intensity of the production process. In turn, TRPM8 activation is increased by the increase of mitochondrial ROS production and then the prostate cancer cells are killed by the TRPM8 channel-induced overproduction of intracellular ROS, apoptosis and Ca^2+^ entry.

As a sulfur containing substance, GSH is containing sulfur groups and it is a member of thiol cycles^2,28^. Oxidation of thiol redox system and cysteine groups in cancer cells have the main role in the activation of thiol group containing TRP channels such as TRPA1, TRPM8 and TRPV1. Intracellular cysteine suppression reduced tumor growth in prostate cancer cells^1,29^. In the current study, the GSH treatment inhibited the oxidative stress and ADPR-induced TRPM8 activation through supporting the thiol cycle antioxidants such as GSH and GSH-Px in the cell line. Similarly, the protective role of GSH treatment on the oxaliplatin-induced TRPA1 activation in mouse dorsal root ganglion (DRG) neurons was reported by Materazzi *et al*.^29^. In addition, it was recently reported that redox-sensitive TRPV1, TRPC1, TRPM2, and TRPM7 channels are inhibited in human hepatoma cell line^30^ and rat DRG neurons^5,24^ by GSH and N acetyl cysteine.

In conclusion, our data clearly show that oxidative stress and ADPR stimulus increased TRPM8-mediated responses, including an increase of intracellular Ca^2+^ and mitochondrial ROS sensitive-apoptosis in the Du 145^M8^ and HEK293^TM8^ cells. However, these responses were attenuated by the treatment with the ROS scavenger GSH and TRPM8 blockers (ACA and CPZ). All together, these data support the hypothesis that oxidative stress is able to induce functional changes in the prostate cancer cell TRPM8 channel signaling and suggest that the killing the prostate cancer cells is susceptible to oxidative stress, with possible implications for treatment of prostate cancer.

## Methods

### Cell lines

Human prostate (Du 145^M8^) cancer cells were purchased from ATCC (Manassas, VA, USA), although HEK293 cells were obtained from the Şap Institute of Agriculture and Animal Ministry of Turkey (Ankara, Turkey). The cells were cultured in a medium consisting of 90% Dulbecco’s modified Eagle’s medium (DMEM, Invitrogen, Istanbul, Turkey), 10% fetal bovine serum (FBS, Gibco, Istanbul, Turkey), and 100 μg/ml streptomycin + penicillin (100 U/ml) combination (Biochrom, Berlin, Germany) and the appropriate supplements, including 100 μg/ml sodium pyruvate (Sigma-Aldrich, Istanbul, Turkey) as suggested by the supplier in a humidified atmosphere in 5% CO_2_ at 37 °C. The cells were tested within 24 hours after plating onto the coverslips. The cells were tested within 24 hours after plating onto the coverslips. Then the cells were counted by using an automatic cell counter (Casy Modell TT, Roche, Germany). In plate reader and patch-clamp analyses, the cells were seeded in 6 flasks at a density of 1 × 10^6^ cells per flask (filter cap, sterile, 260 ml, 80 cm²) (Thermo Fischer Sci. Inc., Istanbul Turkey). In confocal microscope analyses, the cells were seeded in 35 mm glass bottom dishes (Mattek Corporation Inc., Ashland, MA, USA).

### Transfection of HEK293

Transient transfections of HEK293 cells with the 2 μg cDNAs of human TRPM8 (hTRPM8 and a gift from Dr. Simon Hebeisen, B’SYS GmbH, Witterswil Switzerland) were performed according to the manufacturer’s instructions (B’SYS GmbH). For control experiments, 2 μg of wild type TRPM8 empty vector hTRPM8 (C-terminal FLAG tag) plasmid (OriGene Technologies, Istanbul, Turkey) was used for 24 hours using Lipofectamine 2000 (Invitrogen; Istanbul, Turkey. The transfected HEK293 cells (HEK293^TM8^) seeded on glass coverslips at a suitable dilution and were maintained for 24 h in an incubator at 37 °C and 5% CO_2_. Then, patch-clamp, Fura-2 and laser confocal microscope experiments were carried out with cells visibly positive for EGFP.

### Generation of the TRPM8 Knock out Du 145 (Du 145^M8KO^) cell line

Wild type Du 145^M8^ cells were transduced with lentivirus produced as described in a previous study^18^.

### Testing the TRPM8 in the Du 145^M8^ and Du 145^M8KO^ cell lines

Before starting the experiments we tested presence of TRPM8 in the Du 145^M8^ but not in Du 145^M8KO^. Menthol results of TRPM8 were indicated in the current study. Cold exposure to Du 145^M8^ and Du 145^M8KO^ cells in patch-clamp experiments were performed by slice mini bath chamber with controller type as described in a recent study^11^. The TRPM8 is also activated in the Du 145^M8^ but not in Du 145^M8KO^ by cold^18^.

### Determination of intracellular free calcium ion ([Ca^2+^]_i_) concentration in the non-transfected (HEK293) and transfected human HEK293 (HEK293^TM8^) cells, and calcium imaging in Du 145^M8^ and Du 145^M8KO^ cells

The [Ca^2+^]_i_ concentrations in the HEK293^TM8^ and HEK293 cells were monitored using Fura-2-AM as described in a previous study^23^. HEK293^TM8^ and HEK293 grown in 96 well plates, were incubated with Fura-2-AM (4 µM) in phosphate buffer for 45 min at 37 °C in the dark. The groups were exposed to the stimulations in a water-jacketed cuvette (37 °C) with continuous magnetic stirring. Fluorescence was detected by using a Carry Eclipse Spectrofluorometer (Varian Inc, Sydney, Australia). The fluorescence at 505 nm was measured at 1 second intervals after excitation at 340 nm and 380 nm, respectively. Calculation of the [Ca^2+^]_i_ concentrations was described in the previous study^22^, assuming a Kd of 155 nM. The [Ca^2+^]_i_ concentrations in the cells were recorded by using the integral of the rise in [Ca^2+^]_i_ for 160 seconds after the addition of H_2_O_2_ (1 mM) and capsazepine (CPZ and 0.1 mM) as TRPM8 blocker^31^. The [Ca^2+^]_i_ concentration is expressed as nanomolar (nM) taking a sample every second as previously described^23^.

For imaging Du 145^M8^ and Du 145^M8KO^ cells, the cells were analyzed by using Ca^2+^ indicator florescent dye (Fluo-3, Calbiochem, Darmstadt, Germany) in the dark. The Fluo-3 is a single wavelength excitation and emission dye that excited by a 488 nm argon laser from the confocal microscope^32^. The cells were treated with TRPM8 antagonist (ACA and 25 μM) to inhibit Ca^2+^ entry before stimulation of TRPM8 (CHPx and 1 mM). Fluorescence emission of the cells was inspected with a plan Apo 40×/0.2 immersion objective on a confocal microscope (LSM 800, Zeiss, Ankara, Turkey) at 515 nm. Intracellular fluorescence intensities of 10 cells were analyzed in the confocal microscope before CHPx stimulations by ZEN program. Ca^2+^concentration (1.2 mM) and content of the extracellular buffer were described in a previous study^11^. Results of a recent study expressed the importance of TRPM8 on the Ca^2+^ release from intracellular organelles in the prostate cancer cells^33^. For the clarifying importance of Ca^2+^ release from the intracellular organelles through TRPM8 activation we used calcium-free extracellular buffer. In the experiments where calcium-free medium was required, Ca^2+^ was omitted and 2 mM of the chelator EGTA was added.

Manufacturers and preparations of the ADPR, CPZ, menthol, and ACA were described in the previous studies^11,18,23^. The CHPx were dissolved in the extracellular buffer with and without Ca^2+^ (1.2 mM).

### Electrophysiology

Whole-cell voltage clamp recording was taken from the Du 145^M8^, Du 145^M8KO^ HEK293^TM8^ and HEK293 cells (EPC10 patch-clamp set, HEKA, Lamprecht, Germany). We used standard extracellular bath and pipette solutions as described in previous studies^11,18,23^. Holding potential of the patch-clamp analyses in the cell was −60 mV. The current-voltage (I–V) relationships were obtained from voltage ramps from −150 to +150 mV applied over 200 milliseconds. All experiments were performed at room temperature (22 ± 2 °C).

We also performed single cell record experiments in the HEK293^TM8^ cell as described in a previous study^19^.

In the whole cell and single cell experiments, TRPM8 was intracellularly gated by ADPR (1 mM), and the channels were extracellularly blocked by ACA (25 μM). In recent studies, we observed inhibitory role of intracellular GSH (2 mM) on the oxidative stress dependent activations of TRPM2 and TRPV1 channels^11,14,23^. Hence, the TRPM8 channels in some path-clamp experiments were treated with the intracellular GSH. The maximal current amplitudes (pA) in the Du 145 and HEK293 cells were divided by the cell capacitance (pF), a measure of the cell surface. Values of current density were expressed as pA/pF in the patch-clamp experiments.

### Assay of cell viability

Cells were plated in 48-well plates, incubated after treatment with CHPx (1 mM) and ACA (25 μM). Number of viable cell was determined using the 3-(4,5-dimethylthiazol-2yl)-2,5- diphenyl tetrazolium bromide colorimetric (MTT) colorimetric assay as described previously^34^. Absorbance in the spectrophotometer (UV-1800) was read at 570 nm. A total of 3 experiments (n = 3) was performed for the cell viability assay. The data are presented as fold-increase over the pretreatment level.

### Assay of apoptosis, caspase 3 and 9 activities

For the apoptosis spectrophotometric analysis apoptosis, we used a commercial kit and the analyses were performed according to the instructions provided by Biocolor Ltd. (Northern Ireland) and elsewhere^34^.

The determinations of caspase 3 and 9 activities were based on a method previously reported^35,36^ with minor modifications. Caspase 3 (N-acetyl-Asp-Glu-Val-Asp-7-amido-4-methylcoumarin) and 9 (N-acetyl-Leu-Glu- His-Asp-7-amino-4-methylcoumarin) substrates were purchased from Bachem (Bubendorf, Switzerland) and cleavages of the substrates were measured with a microplate reader (Infinite pro200; Tecan Austria GmbH, Groedig, Austria) with excitation wavelength of 360 nm and emission at 460 nm. The data were calculated as fluorescence units/mg protein and presented as fold-increase over the pretreatment level (experimental/control). A total of 3 experiments were performed for the caspase and apoptosis assays.

### Detection of intracellular reactive oxygen species (ROS) level

Dihydrorhodamine- 123 (DHR 123) as a non-fluorescent and non-charged dye can easily diffuse across membranes^34,35^. The Du 145^M8^ and Du 145^M8KO^ cells were washed 1xPBS and they were incubated in DHR123 (1 μl/ml) (Santa Cruz Biotechnology, Inc. Texas USA) at 37 °C in the dark for 30 min. The fluorescence intensity of the oxidized product (Rh123) was measured in the microplate reader (Infinite Pro200). Excitation and emission wavelengths were 488 and 543 nm, respectively^32^. The data are presented as fold-increase over the pretreatment level.

In imaging the ROS production in HEK293^TM8^ and HEK293 cells, the intracellular oxidative stress was monitored by DHR123 (514 nm excitation, 570 emission)^36^. After exposed to indicated treatments, they were incubated in culture medium containing 1 μM DHR123 for 30 min at 37 °C in the dark. Cells were washed and maintained with the phosphate buffer before images were captured using a ZEN Program Imaging System. Fluorescence intensity in 15 μm^2^ of each cell as arbitrary unit was measured by using ZEN program and analyzed using Image J/Imaris software. The results of JC1 and DHR123 were expressed as the mean fluorescence intensity as arbitrary unit /cell.

### Measurement of mitochondrial membrane potential (ΔΨm)

5,5′,6,6′-Tetrachloro-1,1′,3,3′-tetraethylbenzimidazolylcarbocyanine iodide (JC1, Molecular Probes, Eugene, OR, USA) floresecen dye has been using for measurement of ΔΨm level^36^. Hence, we used the dye in the current study for measurement of ΔΨm level. The green (excitation; 485 nm and emission; 535 nm) and red (excitation; 540 nm and emission; 590 nm) JC1 signals were measured in the cell line as described in a previous study^34^. Fluorescence changes were analyzed using the microplate reader (Infinite Pro200). The data are presented as the fold-increase over the pretreatment level.

In imaging of mitochondrial membrane depolarization, the HEK293^TM8^ cells were re-suspended in 0.2 ml of phosphate buffer with calcium and then incubated with JC1 (5 μl) dye solutions for 30 min at 37 °C in the dark. The samples were then analyzed by the laser confocal microscopy. JC1 (505 nm excitation, 535 emission) was excited with a diode laser at 488 nm, an Argon laser at 488 nm^36^. Fluorescence intensity in 15 μm^2^ of each cell as arbitrary unit was measured by using ZEN program and analyzed using Image J/Imaris software. The results of JC1 were expressed as the mean fluorescence intensity as arbitrary unit/cell.

### Annexin V-FITC assay by laser confocal microscope

The protective effects of DTX-induced apoptosis were determined by the laser confocal microscope (LSM-800) using the Annexin V (FITC) dye as described in the manufacturer’s guidelines (Santa Cruz). Briefly, the Annexin V apoptosis detection Kit utilizes FITC-conjugated Annexin V protein for detection of cells undergoing apoptosis. Annexin V FITC binds to the membranes of apoptotic cells, displaying a green characteristic staining pattern which was viewed by the laser confocal microscope (LSM-800).

At the end of the H_2_O_2_ treatment, the HEK293^TM8^ cells were washed twice with phosphate-buffered saline. The cells were re-suspended in 0.2 ml of extracellular buffer and then loaded with Annexin V-FITC (1 μl) for 15 min at room temperature in dark. The samples were then analyzed by the laser confocal microscopy fitted with a 40× oil objective. The fluorescence intensity of each cell as arbitrary unit was measured by using ZEN program and analyzed using Image J/Imaris software. The results of Annexin V-FITC were expressed as the mean fluorescence intensity as arbitrary unit /cell.

### Statistical analyses

All data were represented as means ± standard deviation (SD). Statistical analysis was performed with SPSS Version 18.0 statistic software package (Chicago, Illinois, USA). P value as ≤0.05 was considered to indicate a statistically significant. Presence of significance was once detected by LSD test. Then, comparisons between groups for finding levels of p values were performed with analysis of non-parametric Mann Whitney U test.
